# Supplementary material for: An in vivo probabilistic atlas of the human locus coeruleus at ultra-high field
Source: Neuroimage. 2021 Jan 15;225:117487. doi: 10.1016/j.neuroimage.2020.117487 (PMC7779564; doi:10.1016/j.neuroimage.2020.117487)
Supplement: Supplementary file 1 [file mmc1.docx]

**Supplementary Materials**

**SNR and CNR comparisons between single and averaged MT images**

To demonstrate the effect of averaging method in improving signal sensitivity and reliability, the SNR and the CNR of the LC are calculated using the same equations in the main text for single and averaged MT images. All three MT images acquired for each participant were averaged then compared with either first 2 repeats (R1 and R2) or across all 3 runs (R1, R2 and R3. The values from single and averaged images were subjected to a repeated measures ANOVA with Bonferroni corrected post-hoc comparisons for testing the difference. The test-retest reliability of the MT sequence was tested for both SNR and CNR using Intraclass Correlation Coefficient (ICC) with the two-way mixed effects model (Koo and Li, 2016).

As shown in Figure S1, some individual variations can be observed across different MT scans. The overall reliability across three scans was good for both SNR (ICC=0.67) and CNR (ICC=0.65). Results from repeated measures ANOVA showed that both SNR (F(4,208)=85.25, p<0.001) and CNR (F(4,208)=86.35, p<0.001) are significantly different between single and averaged MT images. The advantage of image averaging has been confirmed with post-hoc tests where both SNR and CNR of the LC were higher for averaged images than these in single MT images (p_bonf_<0.001 for all comparisons between averaged and single image). Averaging across 3 runs can provide higher CNR measurement for the LC than 2 average (p_bonf_<0.001) whereas the SNRs between the two variations of averaging methods were similar (p_bonf_=0.12). Image quality and the LC contrast can also be improved visually after averaging as evidenced in Figure S2.


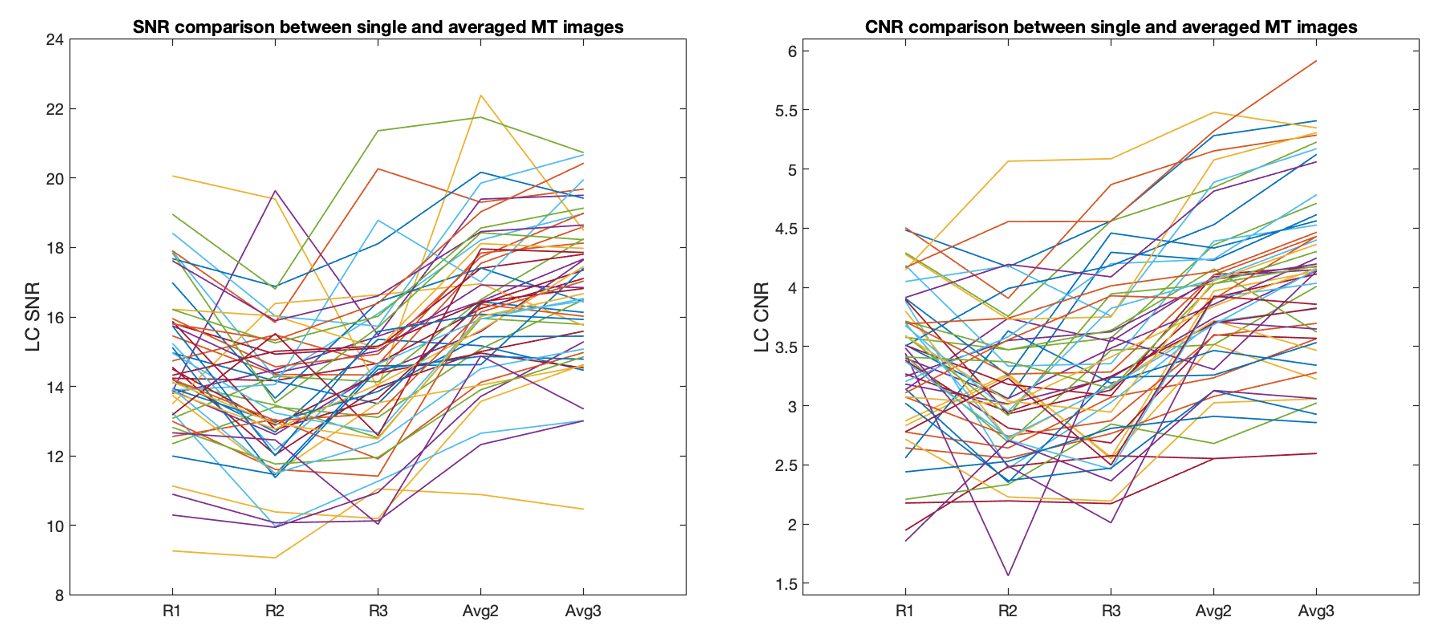


Figure S1. SNR and CNR comparisons of the LC between single and averaged MT images. Each line indicates the data from individual subjects. (R1 = 1^st^ run, R2 = 2^nd^ run, R3 = 3^rd^ run, Avg2 = averaged image with 2 runs, Avg3 = averaged image with 3 runs).

**
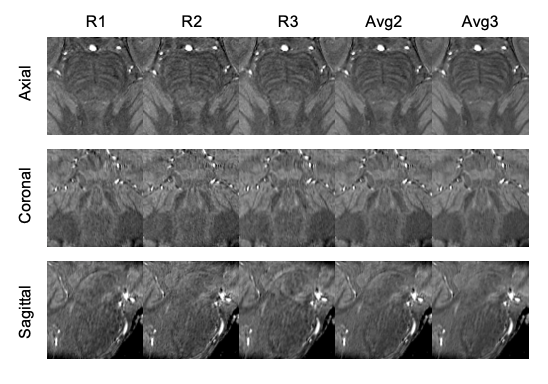
**

Figure S2. Multi-planar views of co-registered MT-on images in each run of the sequence (R1, R2 and R3) and averaged using the first two runs (Avg2) or all 3 runs (Avg3) from a single subject.

**Age effect on LC metrics**

The linear regression model was used to test if age significantly predicts LC CNR and volume with both frequentist and Bayesian inferences. The dependent variables included CNRs for both hemispheres extracted from voxels with peak intensities per slice (peak) or using 5% probability (5% prob) and 25% probability (25% prob) 7T LC atlases. The age effect on LC volumes derived from automated segmentation was also tested. The same model was re-examined in an older subgroup (age > 60 years old) in consistent with previous studies (Liu et al., 2019). In addition, we also tested if age interacted with gender.

We did not find any statistically significant effects of age on LC contrast or volume, for either the whole structure or specific subregions (Table S1). There were also no statistically significant interaction effects between age and gender on LC contrast (Table S2).

Table S1 Statistical results of the linear regression models for age effect on LC CNRs

|  |  | **Age (whole group, n = 53)** | | | **Age (> 60, n = 43)** | | |
| --- | --- | --- | --- | --- | --- | --- | --- |
|  |  | *Frequentist* | *Bayesian* | *R^2^* | *Frequentist* | *Bayesian* | *R^2^* |
| **Whole  LC CNR** | **Peak** | F(1,52)=2.328, *p*=0.133 | BF_10_=0.716 | 0.044 | F(1,42)=1.141, *p*=0.292 | BF_10_=0.473 | 0.027 |
|  | **5% prob** | F(1,52)=1.251, *p*=0.269 | BF_10_=0.462 | 0.024 | F(1,42)=0.391, *p*=0.535 | BF_10_=0.351 | 0.009 |
|  | **25% prob** | F(1,52)=1.556, *p*=0.218 | BF_10_=0.523 | 0.03 | F(1,42)=0.564, *p*=0.457 | BF_10_=0.376 | 0.014 |
|  | **Volume** | F(1,52)=1.875, *p*=0.177 | BF_10_=0.596 | 0.035 | F(1,42)=0.726, *p*=0.399 | BF_10_=0.401 | 0.017 |
| **Rostral  LC CNR** | **Peak** | F(1,52)=2.174, *p*=0.147 | BF_10_=0.673 | 0.041 | F(1,42)=0.628, *p*=0.433 | BF_10_=0.386 | 0.015 |
|  | **5% prob** | F(1,52)=0.720, *p*=0.400 | BF_10_=0.371 | 0.014 | F(1,42)=0.218, *p*=0.643 | BF_10_=0.327 | 0.005 |
|  | **25% prob** | F(1,52)=0.968, *p*=0.330 | BF_10_=0.411 | 0.019 | F(1,42)=0.329, *p*=0.569 | BF_10_=0.342 | 0.008 |
|  | **Volume** | F(1,52)=1.137, *p*=0.291 | BF_10_=0.441 | 0.022 | F(1,42)=0.610, *p*=0.439 | BF_10_=0.383 | 0.015 |
| **Central  LC CNR** | **Peak** | F(1,52)=1.994, *p*=0.164 | BF_10_=0.626 | 0.038 | F(1,42)=1.814, *p*=0.185 | BF_10_=0.618 | 0.042 |
|  | **5% prob** | F(1,52)=1.227, *p*=0.273 | BF_10_=0.458 | 0.023 | F(1,42)=0.258, *p*=0.614 | BF_10_=0.333 | 0.006 |
|  | **25% prob** | F(1,52)=1.952, *p*=0.168 | BF_10_=0.615 | 0.037 | F(1,42)=0.626, *p*=0.433 | BF_10_=0.386 | 0.015 |
|  | **Volume** | F(1,52)=1.783, *p*=0.188 | BF_10_=0.574 | 0.034 | F(1,42)=0.596, *p*=0.445 | BF_10_=0.381 | 0.014 |
| **Caudal  LC CNR** | **Peak** | F(1,52)=2.156, *p*=0.148 | BF_10_=0.668 | 0.041 | F(1,42)=0.742, *p*=0.394 | BF_10_=0.404 | 0.018 |
|  | **5% prob** | F(1,52)=1.748, *p*=0.192 | BF_10_=0.566 | 0.033 | F(1,42)=0.984, *p*=0.327 | BF_10_=0.445 | 0.023 |
|  | **25% prob** | F(1,52)=1.368, *p*=0.248 | BF_10_=0.485 | 0.026 | F(1,42)=0.934, *p*=0.339 | BF_10_=0.436 | 0.022 |
|  | **Volume** | F(1,52)=2.898, *p*=0.095 | BF_10_=0.901 | 0.054 | F(1,42)=0.874, *p*=0.355 | BF_10_=0.426 | 0.021 |

Table S2 Age effect on LC CNRs in male and female subjects

|  |  | **Age (whole group, n = 53)** | | |
| --- | --- | --- | --- | --- |
|  |  | *Frequentist* | *Bayesian* | *R^2^* |
| **Female (n=24)** | **LC CNR (Peak)** | F(1,23)=2.920, *p*=0.102 | BF_10_=1.000 | 0.117 |
|  | **LC CNR (5% prob)** | F(1,23)=2.250, *p*=0.148 | BF_10_=0.831 | 0.093 |
|  | **LC CNR (25% prob)** | F(1,23)=2.144, *p*=0.157 | BF_10_=0.801 | 0.089 |
| **Male  (n=29)** | **LC CNR (Peak)** | F(1,28)=0.152, *p*=0.699 | BF_10_=0.369 | 0.006 |
|  | **LC CNR (5% prob)** | F(1,28)=0.005, *p*=0.945 | BF_10_=0.349 | <0.001 |
|  | **LC CNR (25% prob)** | F(1,28)=0.062, *p*=0.805 | BF_10_=0.357 | 0.002 |

LC CNRs were estimated for the whole structure with corresponding methods.

**References**

KOO, T. K. & LI, M. Y. J. J. O. C. M. 2016. A guideline of selecting and reporting intraclass correlation coefficients for reliability research. 15**,** 155-163.

LIU, K. Y., ACOSTA-CABRONERO, J., CARDENAS-BLANCO, A., LOANE, C., BERRY, A. J., BETTS, M. J., KIEVIT, R. A., HENSON, R. N., DUZEL, E., HOWARD, R., HAMMERER, D. & CAM-CAN 2019. In vivo visualization of age-related differences in the locus coeruleus. *Neurobiology of Aging,* 74**,** 101-111.
